# In Vitro Study on Anti-Hepatitis C Virus Activity of *Spatholobus suberectus* Dunn

**DOI:** 10.3390/molecules21101367

**Published:** 2016-10-15

**Authors:** Shao-Ru Chen, An-Qi Wang, Li-Gen Lin, Hong-Cong Qiu, Yi-Tao Wang, Ying Wang

**Affiliations:** 1 State Key Laboratory of Quality Research in Chinese Medicine and Institute of Chinese Medical Sciences, University of Macau, Avenida da Universidade, Taipa, Macao SAR 999078, China; shalleychen1@gmail.com (S.-R.C.); yb37510@umac.mo (A.-Q.W.); ligenl@umac.mo (L.-G.L.); 2 Guangxi Institute of Traditional Medical and Pharmaceutical Sciences and Guangxi Key Laboratory of Traditional Chinese Medicine Quality Standards, Nanning 530022, China; qiuhongcong@hotmail.com

**Keywords:** HCV, *S. suberectus* extraction, anti-HCV therapy, tannin

## Abstract

Hepatitis C virus (HCV) infects 200 million people worldwide, and 75% of HCV cases progress into chronic infections, which consequently cause cirrhosis and hepatocellular carcinoma. HCV infection is treated with currently considered standard drugs, including direct anti-viral agents (DAAs), alone or in combination with peginterferon-α plus ribavirin. However, sustained viral responses vary in different cohorts, and high costs limit the broad use of DAAs. In this study, the ethanol and water extracts of 12 herbs from Lingnan in China were examined in terms of their inhibitory effect on HCV replication. Among the examined extracts, *Spatholobus suberectus* ethanol extracts suppressed HCV replication. By comparison, Extracts from *Fructus lycii*, *Radix astragali* (root), *Rubus chingii Hu* (fruit), *Flos chrysanthemi Indici* (flower), *Cassia obtusifolia* (seed), *Lonicera japonica* Thunb (flower), *Forsythia suspense* Thunb (fruit), *Poria cocos* (sclerotia), *Carthamus tinctorius* L. (flower), *Crataegus pinnatifida Bge.* (fruit), and *Leonurus japonicas* Houtt. (leaf) extracts failed to show a similar activity. Active *S. suberectus* fractions containing tannins as the major component also inhibited the in vitro translation of HCV RNA. The combination treatments of single compounds, such as epigallocatechin gallate and epicatechin gallate, were not as potent as crude *S. suberectus* fractions; therefore, crude *S. suberectus* extract may be a potential alternative treatment against HCV either alone or in combination with other agents.

## 1. Introduction

Hepatitis C virus (HCV) is a hepacivirus with a positive-sense single-strand RNA genome that belongs to the Flaviviridae family. HCV currently infects 200 million people worldwide [1]. More than 75% of HCV infections eventually lead to chronic infections, which then progress to cirrhosis and hepatocellular carcinoma [2,3,4]. HCV infection-associated morbidity and mortality are predicted to increase in the next 20 years, although the prevalence of new infections has declined [5,6,7]. The prevalence of HCV infections in North America, Western Europe, and Australia is low; by contrast, the prevalence of such infections in Africa and Asia, especially in the coastal regions and border provinces in China is high [8,9,10,11]. Without effective vaccines, primary prevention against HCV infections focuses on reducing risks by promoting safe injection practices for drug users and by implementing quality control of blood for transmission [11,12].

After entering hepatocytes, the HCV genome translates into structural and non-structural (NS) proteins, which include core protein, envelope proteins (E1 and E2), p7, and NS proteins from NS2 to NS5B [13]. Translation from viral genomes to proteins is the most important step in HCV replication. Therefore, NS proteins play an important role in HCV replication and serve as primary targets for anti-viral therapy.

The current treatments for HCV infection include the combination of pegylated interferon α (pegIFNα) injection and oral ribavirin administration for 24 or 48 weeks [14]. The respective response rates of genotypes 1, 2, and 3 for pegIFNα/ribavirin therapy are 35%, 74%, and 62% because of various baseline viral loads, different genotypes, and T helper 1 immune reaction [15,16,17,18]. However, pegIFNα/ribavirin therapy is associated with severe side effects, such as psoriatic arthritis and hemolytic anemia [19]. In addition to those combined pegIFNα/ribavirin treatment, direct-acting antiviral (DAAs) agents, including NS3/4A protease inhibitors boceprevir, simeprevir, telaprevir, NS5A phosphatase inhibitor daclatasvir, and RNA polymerase inhibitor sofosbuvir, have been approved for targeted therapy against viral NS proteins [20,21,22,23]. DAAs, together with pegIFNα/ribavirin, exhibit high sustained viral response (SVR) rates with a broad genotype coverage [24]. For example, simeprevir combined with pegIFNα/ribavirin increases the SVR rates to approximately 80% [22]. Nevertheless, high costs and low SVR rates in genotype 3 restrict the broad application of DAAs [20]. Therefore, additional therapeutic strategies are necessary to manage HCV infection, especially for high-risk populations with relatively low income.

Herbal medicine is a rich resource for drug discovery and development. Clinical medications are directly or indirectly derived from medicinal plants [25,26]. For instance, two multiple-component herb extracts, namely, Veregen^®^ (polyphenon E) from green tea, and Fulyzaq from *Croton lechleri* sap, have been approved by the Food and Drug Administration of USA to treat genital warts and anti-HIV nucleoside- and nucleotide analog-associated diarrhea, respectively [27,28]. Herb extracts and phytochemicals with anti-viral activities have also been extensively investigated. For example, mulberry (*Morus alba*, Ma) juice and its major polyphenol component (cyanidin-3-glucoside and cyanidin-3-rutinoside) inhibit the internalization and replication of murine norovirus-1 and the adherence or internalization of feline calicivirus-F9 [29]. Clemastanin B, the major lignan from *Isatis indigotica* root (*Radix isdtidis*), specifically inhibits different subtypes of human and avian influenza viruses at concentrations below 1 mg/mL [30,31,32]. *Ilex asprella* (Hook. & Arn.) Champ. Ex Benth., a folk medicine used in Southern China, relieves acute respiratory distress syndrome induced by H1N1 virus through the inhibition of viral replication [33].

According to traditional Chinese medicine theory, viral infection is regarded as internal heat. As such, various traditional herbal teas and soups cooked with heat-reducing herbs are served as routine diets in hot and humid regions in China. For example, *Spatholobus suberectus*, which is also known as Ji Xue Teng (JXT) in the Chinese Pharmacopoeia or *S. suberectus Dunn* vine stem, has been widely used in traditional Chinese and folk medicine to treat blood deficiency, irregular menstruation, and rheumatalgia [34]. *S. suberectus* has also been applied to manage blood stasis syndrome, menoxenia, and rheumatism [34]. *S. suberectus*, a “master drug” in the Chinese herbal medicine formula “compound 861”, reduces liver enzymes and alleviates fibrosis in HBV patients after 24 weeks of treatment [35,36]. Phytochemical studies have further revealed that *S. suberectus* mainly contains polyphenols, including chalcones, flavanones, flavanols, and isoflavonoids [37,38,39]. *S. suberectus* treatment exhibits antiviral activities on coxsackie virus B3 and enteroviruses [40,41,42]. However, the anti-HCV activity of *S. suberectus* has yet to be reported. In our study, the anti-HCV activity of commonly used herbs, including those that promote blood circulation and dispel heat from the region, was initially examined. Among the examined herbs, *S. suberectus* Dunn extract exhibited a potent anti-HCV activity. In particular, 50%–75% ethanol-eluted fractions of *S. suberectus* aqueous extract induced a strong anti-HCV activity and inhibited the translation of HCV RNA. Tannin enriched in 50%–75% ethanol fractions of *S. suberectus* extracts exhibited the highest anti-HCV activity. By contrast, none of the combinations of different compounds, such as epigallocatechin gallate (EGCG) or epicatechin gallate (ECG), significantly inhibited HCV replication.

## 2. Results

**Figure 1:**
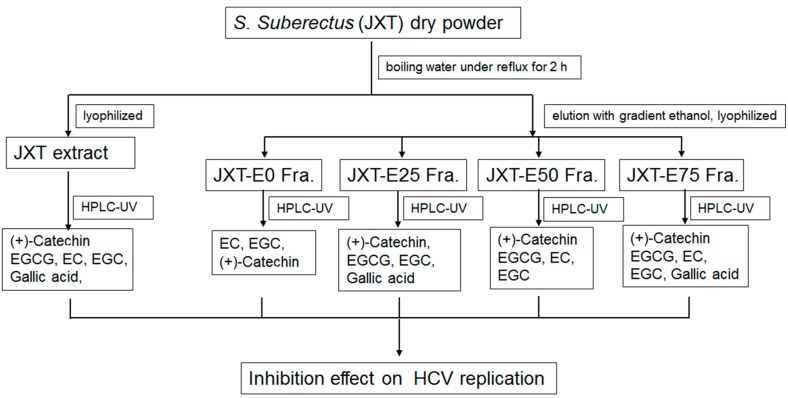
Extraction scheme of *S. suberectus*. Ethanol fractions were labeled as E followed by the percentage of ethanol used; for example, JXT-E25 means 25% ethanol eluted fraction of *S. suberectus* (JXT).

According to traditional Chinese medicine theory, viral infections are considered inflammation and internal heat reduction. Therefore, we aimed to determine herbs commonly used as HCV treatment. We selected 12 herbs, including tonic drugs, heat-dispelling drugs, and blood-circulation-promoting drugs (Table 1), which target the liver channel. The herbs were extracted with water and fractionated with different percentages of ethanol (Figure 1 showing *S. suberectus* as an example).

The anti-HCV activity and cytotoxicity of these herb extracts were initially determined against HCV genotype 1b replicon cells. *S. suberectus* extracts (100 μg/mL; Table 1) inhibited HCV replication. The EC_50_ of the active extracts against HCV was then determined by using different fractions of *S. suberectus.* The EC_50_ of *S. suberectus* extracts, except the aqueous extract (JXT-E0; Table 2) against HCV was approximately 1 μg/mL. Moreover, 100 μg/mL *S. suberectus* extract did not elicit cytotoxic effects except the aqueous extract of JXT-E0 (Table 2). *S. suberectus* fractions were less active than DAAs and IFNα-2a against HCV replication (Table 2).

**Figure 2:**
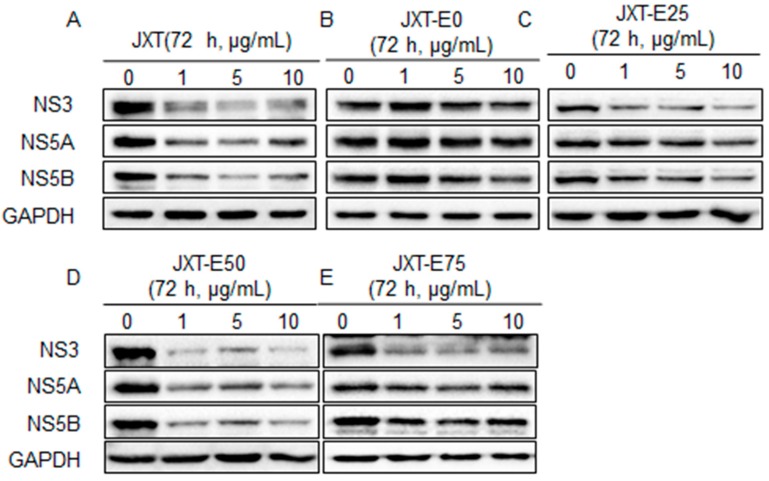
Expression levels of HCV NS proteins with the treatment of (**A**) JXT; (**B**) JXT-E0; (**C**) JXT-E25; (**D**) JXT-E50, and (**E**) JXT-E75 extracts. Huh-luc/neo-ET cells were seeded in a 6-well plate, and treated with different concentrations of JXT fractions for 72 h. The cells were collected and lysed, and 30 ng of the total protein was subjected to SDS PAGE and western blot. The expression levels of NS3, NS5A, and NS5B were determined. The expression level of GAPHD was used as the housekeeping control. The results are representative of three independent experiments.

**Figure 3:**
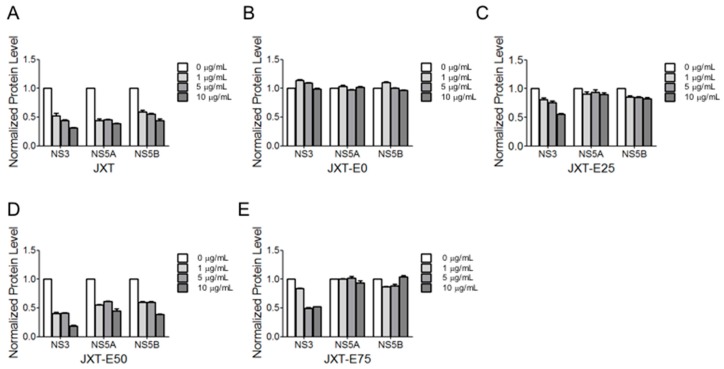
Quantitative results of protein expression levels of NS3, NS5A, and NS5B with treatments of (**A**) JXT; (**B**) JXT-E0; (**C**) JXT-E25; (**D**) JXT-E50; and (**E**) JXT-E75 extracts. The expression levels of NS3, NS5A, and NS5B were normalized to that of GAPDH.

**Figure 4:**
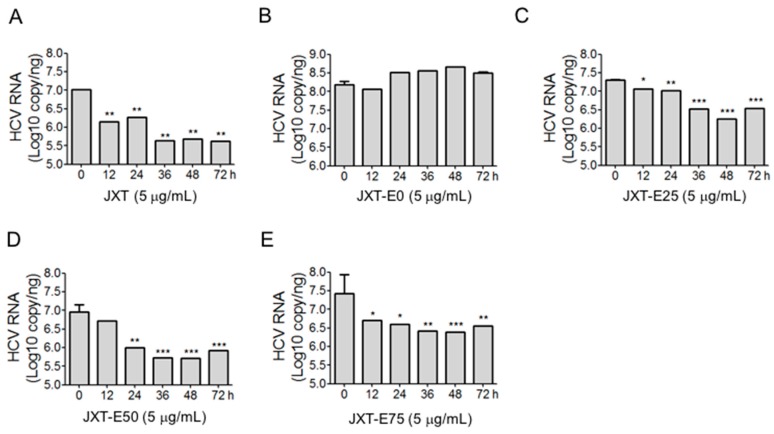
Effect of (**A**) JXT; (**B**) JXT-E0; (**C**) JXT-E25; (**D**) JXT-E50; and (**E**) JXT-E75 extracts on HCV RNA levels. Huh-luc/neo-ET cells were seeded in a 6-well plate and treated with 5 μg/mL of JXT fractions for different time periods. At the end of the treatment, RNA was extracted with Trizol and converted to cDNA. The level of HCV RNA was determined through real-time PCR. The expression level of 18S rRNA was used as a control. The absolute copy number of HCV RNA was determined with the standard curve generated by using the linearized pFK-I389/NS3-3′ plasmid. The results were obtained from three independent experiments and presented as mean ± SD (* *p* < 0.05, ** *p* < 0.01, *** *p* < 0.001 compared with DMSO control). Some of the error bars were too small to be seen.

**Figure 5:**
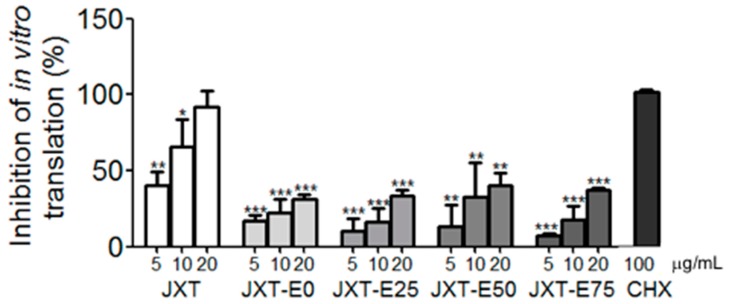
Effects of whole water and different ethanol extracts of JXT, and CHX on the translation of HCV RNA in a rabbit lysis cell-free lysate system. The results were obtained from three independent experiments and presented as mean ± SD (* *p* < 0.05, ** *p* < 0.01, *** *p* < 0.001 compared with DMSO control).

The expression levels of HCV NS proteins with the treatments of different fractions were determined through western blot analysis (Figure 2). Quantitative results of protein expression levels were determined by Image J software and shown in Figure 3. JXT and JXT-E50 treatments (1 μg/mL) suppressed the HCV NS protein expression levels (Figure 2A,D and Figure 3A,D). JXT-E50 treatment also inhibited more than 50% of NS5A and NS5B expression levels, and more than 80% of NS3 expression level than the untreated control did (Figure 2D). Therefore, JXT-E50 treatment was the most potent among all of the tested fractions. JXT-E25 and JXT-E75 treatments (10 μg/mL) decreased the expression of NS3 by approximately 50% without inhibiting the expression levels of NS5A and NS5B (Figure 2C,E and Figure 3C,D). Under the same conditions, JXT-E0 treatment (10 μg/mL) did not change the expression of NS proteins for 72 h (Figure 2B and Figure 3B). To confirm the inhibitory effects of *S. suberectus* extracts on HCV replication, we determined the RNA levels of HCV treated with *S. suberectus* extract through qRT-PCR. JXT, JXT-E25, JXT-E50, and JXT-E75 (5 μg/mL) markedly decreased the copy number of HCV RNA in a time-dependent manner (Figure 4A,C–E). By contrast, JXT-E0 treatment did not inhibit HCV RNA replication under the same conditions (Figure 4B). Among these fractions, 5 μg/mL JXT and JXT-E75 inhibited the expression levels of more than 30% of HCV RNA for only 12 h (Figure 4A,E). JXT-E50 suppressed the expression of approximately 50% of HCV RNA after 24 h of treatment at 5 μg/mL (Figure 4D). JXT-E25 (5 μg/mL) inhibited the HCV RNA expression in a time-dependent manner (Figure 4C). However, JXT-E0 treatment did not inhibit HCV RNA replication under the same conditions (Figure 4B). The translation of HCV RNA to polyprotein is an essential step in HCV replication [1]. Treatments with whole water and different ethanol fractions of JXT inhibited the translation of HCV RNA in the in vitro rabbit lysis system in a dose-dependent manner (Figure 5). Treatment with the translation inhibitor cycloheximide (CHX) was used as a positive control (Figure 5).

**Figure 6:**
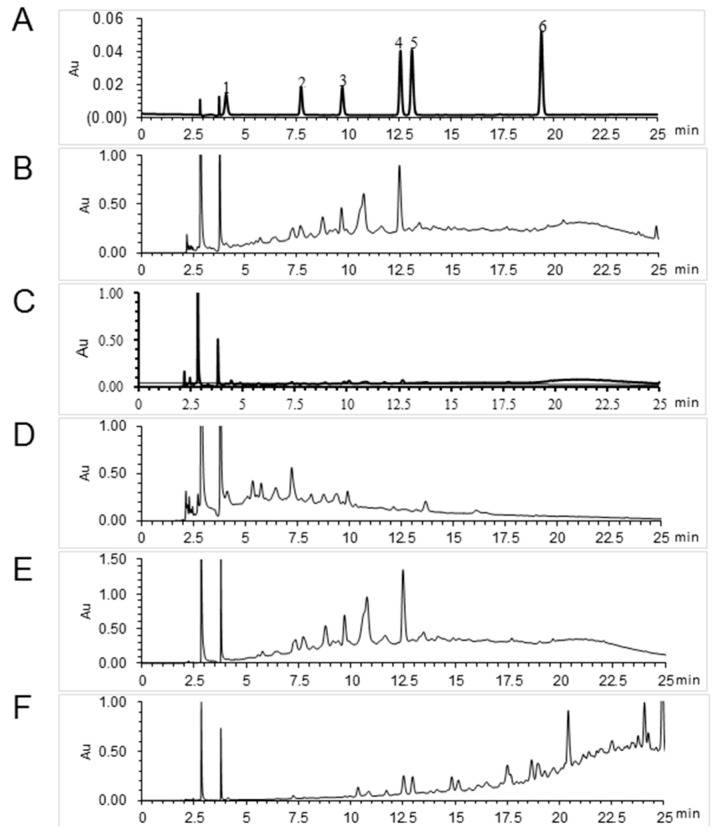
Chromatogram of the representative constituents in the water extract of *S. suberectus* and sub-fractions obtained from macroporous resin with gradient ethanol elution. (**A**) Mixed standard solution of gallic acid (1); epigallocatechin (2, EGC); catechin (3); epicatechin (4, EC); epigallocatechin gallate (5, EGCG), and epicatechin gallate (6, ECG) at 2.50, 21.92, 10.67, 14.25, 7.64 and 11.75 μg/mL, respectively; (**B**) Prepared samples of crude water extracts of *S. suberectus*, and sub-fractions eluted from macroporous resin by (**C**) 0%; (**D**) 25%; (**E**) 50%; and (**F**) 75% ethanol solutions at 41.28, 23.72, 33.46, 33.70, and 31.75 mg/mL, respectively.

**Figure 7:**
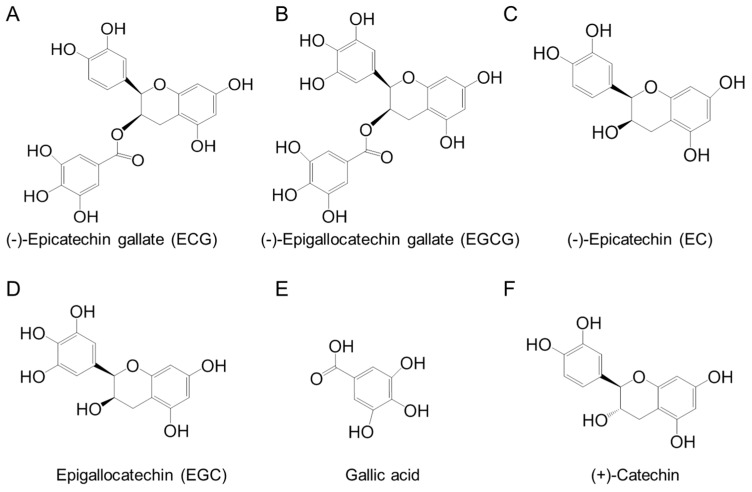
Chemical structures of (**A**) ECG; (**B**) EGCG; (**C**) EC; (**D**) EGC; (**E**) gallic acid; (**F**) (+)-catechin.

**Figure 8:**
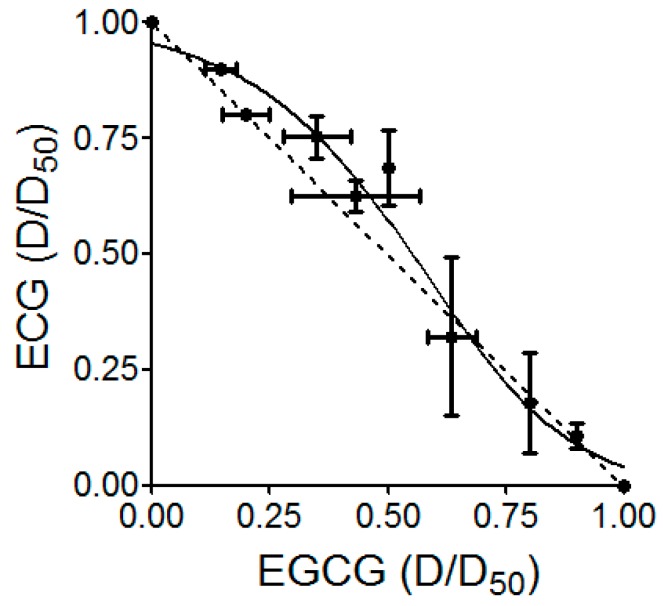
Anti-HCV replication effects under the combined treatment with EGCG and ECG in HCV replicon cells. Huh-luc/neo-ET replicon cells were incubated with different concentrations of EGCG and ECG for 72 h alone or in combination. The ratios of the detected EC_50_ of EGCG and ECG alone or in combination at EC_50_ of single compound were plotted against each other in an isobologram. Each point on the isobologram represents a combined treatment that inhibited 50% of HCV replication (mean ± SD from three separate experiments).

We then identified the major component(s) in the different fractions of *S. suberectus* extract that exhibited anti-HCV activity. We analyzed *S. suberectus* extracts through HPLC to determine the major component(s) in the fractions. Table 3 and Figure 6 revealed the concentrations of the major components in different fractions of *S. suberectus* extracts: gallic acid, epigallocatechin (EGC), catechin, epicatechin (EC), EGCG, ECG (chemical structures shown in Figure 7), and tannins. Tannins appeared as the major components in 25%–75% ethanol eluted fractions (Table 3). Among these components, EGCG and ECG, but not gallic acid, EC, nor (+) catechin (Table 4), exhibited inhibitory effects on HCV (Table 4). Considering the relatively low amounts of ECGC and ECG in JXT-E50 and JXT-E75 (Table 3), we determined the anti-HCV activity by using a combination of different components. The combined treatment of EGCG and ECG exhibited additive anti-HCV activity (Figure 8), whereas the combination of other compounds (Table 3) did not elicit a similar effect (data not shown).

## 3. Discussion

Chronic HCV infection is a major cause of severe liver diseases, including liver fibrosis/cirrhosis and cancer. Despite the recent success of HCV treatments with DAAs alone or in combination with pegIFNα/ribavirin, alternative treatment methods should be further developed for certain patients groups, such as those with genotype 3, renal failure, hepatic decompensating, and liver transplantation [43,44]. In addition, high risk populations with low income have limited access to DAAs because of high costs. Therefore, accessible therapeutic options for HCV infections are still required. Medicinal plants, such as *Detarium microcarpum* and *Phargmanthera capitata* from sub-Saharan African countries; and *Maytrenus ilicifolia* from Brazil are natural resources that provide various novel activities [45] and remarkably inhibit HCV replication [46,47].

HCV genotype 1b is a resilient HCV strain with the highest prevalence associated with end-stage liver diseases [48,49]. Viral infections have been widely treated with herbal extracts [50,51]. We determined 12 commonly used herbs for the anti-HCV activity by using the genotype 1b replicon system. According to traditional Chinese medicine theory, these herbs belong to three different categories, including tonic drugs, heat-dispelling drugs, and blood-circulation-promoting drugs (Table 1). We also summarized the target channels or organs related to their therapeutic activities in accordance with traditional Chinese medicine theory (Table 1). Among the 12 herbs, only *S. suberectus* extracts potently inhibited the activity of HCV genotype 1b and the translation of HCV RNA in vitro (Table 1 and Figure 5). *S. suberectus* extracts, especially 50% ethanol-eluted fraction (JXT-E50), was the most potent (Table 1 and Table 2, and Figure 2, Figure 3 and Figure 4).

Aqueous and methanolic *S. suberectus* extracts inhibit the production of *Dactylogyrus intermedius* in goldfish at concentrations below 10 mg/L [52]. Ethyl acetate *S. suberectus* extract suppress the growth of human osteosarcoma Saos-2 cells in vitro [53]. Aqueous *S. suberectus* extract prevents the platelet-activating factors or ADP-induced aggregation of platelets in platelet-rich plasma dose dependently [54]. Sixteen phenolic bioactive constituents, including flavanols, isoflavonoids, flavanones, and chalcone, have been identified in *S. suberectus* extract [34]. Flavanoes from *S. suberectus* are active against the growth of different cultured human cancer cells [55]. Flavanones from other medicinal plants/herbal medicines possess anti-HCV or -HBV activities [56,57]. For example, flavonol quercetagetin blocks HCV RNA-dependent RNA polymerase activity [57]. We demonstrated that *S. suberectus* extracts, except JXT-E0, exhibited anti-HCV activity (Table 2). Among the ethanol-eluted fractions, JXT-E50 significantly prevented HCV replication, NS protein expression, HCV RNA expression, and HCV RNA translation (Figure 2, Figure 3, Figure 4 and Figure 5). JXT-E25 and JXT-E75 also decreased the expression of NS3 by approximately 50% without inhibiting the expression levels of NS5A and NS5B at as high as 10 μg/mL (Figure 2C,E and Figure 3C,D). The polarity of compounds eluted from different percentages of ethanol fractions is accounted for the different components of each fraction. As a consequence, these compounds exhibit different activities against HCV. Therefore, JXT-E50 was considered the most potent fraction.

In general, curative effects are based on multiple components and multiple targets. Thus far, only a limited number of components have been identified from *S. suberectus* extracts [34]. Gallic acid, one of the components of *S. suberectus*, can inhibit HCV entry and suppress HCV infection in vitro [58]. EGCG, another major component of *S. suberectus*, inhibits the replication of HCV JFH1-GFP chimeric virus, J399EM virus, and HCV entry with EC_50_ of 17.9, 80, and 25 μM, respectively [59]. Catechin also exhibits a potent anti-HCV activity in the HCV-JFH1 viral culture system [60]. However, the concentrations of these compounds required to achieve 50% inhibition of HCV activity were high in previous reports and our study (Table 3). This phenomenon may occur because of the HCV genotype 1b replicon cells used in our study and the HCV genotype 2a JFH-1 isolate examined in previous reports. Therefore, these compounds could not be considered as active components of *S. suberectus* for HCV genoype 1b.

The identification of single active component(s) partly contradicts Traditional Chinese Medicine theory, which postulates that active ingredients and herbs function in combination with all or most of the gradients instead of as isolated single component [61]. Different components in crude herb extracts also interact with one another additively, synergistically, or antagonistically. For example, the polyphenol fraction of green tea extract yields an anti-genital wart activity compared to its single polyphenol components [27]. This combined effect is also observed in herbal formulas; for instance, no single herb achieves a similar anti-inflammatory activity against CPT-11-induced gastrointestinal toxicity as PHY906 formula did in a preclinical study [62]. Moreover, the combination of EGCG and ECG exhibited an additive effect against HCV replication. However, JXT-E50 and JXT-E75 exhibited potency against HCV than other fractions (Table 3 and Table 4, Figure 2, Figure 3 and Figure 4). The main components of JXT-E50 and JXT-E75 are tannins (Table 3). Therefore, our results confirmed that herb extracts exert activity in as a whole instead of as single pure compounds. The pure separation of single component(s) may greatly reduce the activity of herb extracts.

*S. suberectus* is a commonly used herb that can be easily obtained by the Chinese. In our study, *S. suberectus* extracts potently inhibited HCV activity and HCV RNA translation in the genotype 1b replicon culture system without a detectable cytotoxicity. Tannins enriched in the ethanol-eluted fractions of *S. suberectus* extract but no single compound was considered as an active components. Thus, this herb can be utilized as a potential alternative alone or in combination with DAAs, and pegIFNα/ribavirin for the treatment of HCV in high risk populations with relatively low income.

## 4. Materials and Methods

### 4.1. Materials and Reagents

The pFK-I389/NS3-3′ plasmid and HCV genotype 1b (Con l isolate) subgenomic replicon cell line Huh-luc/neo-ET containing a luciferase reporter were provided by Ralf Bartenschlager (University of Heidelberg, Heidelberg, Baden-Württemberg, Germany) [63]. Cell culture media and fetal bovine serum (FBS) were purchased from Life Technologies (Waltham, MA, USA). Boceprevir (Cat. B674500, CAS 394730-60-0), sofosbuvir (Cat. P839643, CAS 1190307-88-0), simeprevir (Cat. S466500, CAS 923604-59-5), telaprevir (Cat. T015650, CAS 402957-28-2), daclatasvir (Cat. D101500, CAS 1009119-64-5) were purchased from Toronto Research Chemicals (Toronto, ON, Canada). IFNα-2A (Cat. Z03003-50) was purchased from Genscript (Piscataway Township, NJ, USA). All chemicals were purchased from Cayman Chemical (Ann Arbor, MI, USA) except for those noted otherwise. All other reagents were purchased from Sigma-Aldrich (Taufkirchen, Germany) except noted otherwise.

### 4.2. Extraction and Isolation of Herbs

All the dried herbs used in this study were purchased from Guangdong Kangmei Pharmaceutical Co., Ltd. (Pu Ning, Guangdong, China). All herbs were identified by Jin-Gui Shen (Shanghai Institute of Materia Medica, Shanghai, China). A voucher was deposited at the herbarium of Institute of Chinese Medical Sciences, University of Macau, Macau, China, (LL-20140602,).The herbs above were ground into powder. One hundred grams of dry powder from each herb was extracted with boiling Mili-Q (Merck Millipore, Billerica, MA, USA) water (500 mL) under reflux for 2 h, and repeated for three times. Then the solution was filtered twice through no. 41 filter paper (Whatman, PA, USA). The filtrate was evaporated to obtain the crude extract. Each crude extract was subjected to column chromatography over macroporous resin and eluted with 0%, 25%, 50%, 75% ethanol (Figure 1). All fractions together with the original crude extract were lyophilized and stored at −20 °C for further use. All extracts were dissolved with DMSO, and a final concentration of 100 μg/mL was obtained by dilution in culture media so that the final concentration of DMSO did not exceed 0.05% of total volume. DMSO was used as control in all following experiments.

### 4.3. Chromatographic Analysis

The chromatographic analysis was performed on an e2695 apparatus (Waters, WI, USA) equipped with a PDA detector. The Waters SunFire™ C_18_ (4.6 mm × 250 mm, 5 μm, Waters) column coupled with a YMC-Triart C18 (4.0 mm × 10 mm, 5 µm, YMC, Kyoto, Japan) guard column was maintained at 35 °C. Samples were eluted with gradient water (0.1% phosphoric acid) (A)-acetonitrile (B) at a flow rate of 1 mL/min and monitored at 273 nm. The gradient elution program was set as follows: 0~15 min, 90%~80% A; 15~55 min, 80%~10% A; 55~56 min, 10%~90% A; 56~63 min, 90% A. The representative chromatograms of standard solution and samples were analyzed by using the Waters Empower system. The content of representative constituents in each sample was calculated by comparing the corresponding peak area with that of the standard solution detected at the same retention time (RT).

Samples were prepared in specified concentration (41.28, 23.72, 33.46, 33.70, and 31.75 mg/mL for JXT, JXT-E0, JXT-E25, JXT-E50 and JXT-E75 respectively) and subsequently filtered by 0.45 μm PTFE filter membrane (Millipore). Then, 10 μL solutions were injected into HPLC column and analyzed through gradient elution.

### 4.4. Determination of Tannins in JXT Extracts

The content of tannins in JXT samples was determined as described in the Chinese Pharmacopeia (2010 version) [64]. In the first step, 0.2 mg/mL of each JXT sample was prepared in water. One milliliter of each sample was introduced into a 25 mL volumetric flask, and mixed with 1 mL phosphomolybdic tungstic acid solution (Sigma-Aldrich, St. Louis, MO, USA) and 3 mL water, followed by the addition of 29% sodium carbonate solution to make up final volume to 25 mL. The reaction solution was mixed thoroughly and allowed to stand for 30 min at room temperature. Water was used as a blank control, and 0.05 mg/mL gallic acid solution was used as standard solution. Absorbance was measured at 760 nm by a microplate reader (1420 Multilabel Counter Victor 3, Perkin Elmer, Wellesley, MA, USA). The content of total phenols was calculated as following: Ct% = (At − A0)/AS × 100, where Ct was the content of total phenols, At, A0 and AS were the absorbance of sample, blank control, and standard solution, respectively. In the second step, 1 mL of each sample was mixed with 0.5 g casein, followed by addition of water to a final volume of 10 mL. The mixture was reacted at 30 °C for 1 h in a shaking water bath. The reaction mixture was filtered after cool to room temperature. Subsequently, 3 mL of filtration was introduced into a 25 mL brown volumetric flask, and reacted with 1 mL phosphomolybdic tungstic acid solution as in the first step. The content of free phenols was calculated as following: Cf% = (At′ − A0′)/AS′ × 100, where Cf was the content of free phenols, At′, A0′ and AS′ were the absorbance of each sample, blank control, and standard solution, respectively. Finally, the content of tannins was calculated as following: C% = Ct − Cf. The results were analyzed based on at least three independent experiments.

### 4.5. Cell Culture Conditions

Huh-luc/neo-ET cells were cultured in High Glucose Dulbecco′s modified Eagle’s medium, supplement with 10% FBS, 1 mM non-essential amino acid, and 250 μg/mL G418 at 37 °C in 95% air and 5% CO_2_ environment.

### 4.6. Anti-HCV Assay on Huh-luc/neo-ET Cell Line In Vitro

The anti-HCV EC_50_ (half maximal effective concentration) of herbal extracts were determined using a luciferase reporter assay with HCV genotypes 1b (Con1) replicon cells free from G418 [65,66]. Luciferase activity was measured with a luciferase assay kit (Promega, Fitchburg, WI, USA) on a SpectraMax M5 multi-mode reader (Molecular Devices, Sunnyvale, CA, USA) according to the manufacturer’s instructions.

### 4.7. Cytotoxicity Analysis

The cytotoxicity of different herbal extract or components was measured by methylene blue assay. Huh-luc/neo-ET cells were seeded at a density of 7 × 10^3^ cells per well in 96-well plate and treated with serial dilution of different herb extracts for 72 h. Cells were fixed and stained with 0.5% methylene blue in 50% ethanol, and then washed with tap water. After air-dry, cells were solubilized with 1% Sarkosyl and cell viability was determined from the extent absorption by spectrophotometric measurements at 595 nm (Spectra Max M5, Molecular Devices).

### 4.8. Western Blot Analysis

Herb extract and DMSO treated Huh-luc/neo-ET cells were harvested, and extracted with RIPA lysis buffer including 1% protease inhibitor (Life Technologies). The expression levels of NS3 (Millipore, MAB8691), NS5A (Millipore, MAB8694), NS5B (Abcam, Cambridge, UK, AB100895), GAPDH (Santa Cruz, CA, USA sc-25778) and were determined by using specific antibodies. All primary antibodies were diluted for 1000 fold with 1% not fat milk. The band intensity was analyzed with Image J (National Institutes of Health, New York City, NY, USA). Results were normalized against the band intensity of GAPDH.

### 4.9. Extraction of Total RNA and Quantitative Real-Time-PCR (qRT-PCR) Analysis

Huh-luc/neo-ET cells were seeded at a density of 1.6 × 10^5^ cells in T25 cm^2^ flask and treated with either DMSO, or herb extracts for different times. At the end of the treatment, total RNA was extracted by Trizol (Life Technologies). One microgram of total RNA was reverse transcribed into cDNA with ProtoScript^®^ II Reverse Transcriptase (New England Biolabs, Ipswich, MA, USA). qPCR was performed in triplicates for all samples using the Mx 3005P qPCR system (Agilent, Santa Clara, CA, USA) with SYBR Master Mix (Bio-Rad, Hercules, CA, USA). Absolute copy number of HCV RNA was determined with the standard curve generated by using the linearized pFK-I389/NS3-3′ plasmid bearing the HCV genome and 18S rRNA was included as a control. The primers were as follows: forward primer 5′-CTTCACGCAGAAAGCGTCTA-3′ and reverse primer 5′-CAAGCACCCTATCAGGCAGT-3′ for HCV; forward primer 5′-GTAACCCGTTG AACCCCATT-3′ and reverse primer 5’-CCATCCAATCGGTAGTAGCG-3′ for 18S rRNA.

### 4.10. Combination Study

Huh-luc/neo-ET cells were seeded at a density of 7 × 10^3^ cells per well in 96-well plates. On the following day, a mixture of different combinations of single compounds, including ECG, EGCG, EC, EGC, gallic acid, and (+) catechin, was applied in a series dilution and kept at a constant ratio. Determination of additive, antagonism, or synergism of combination treatment was calculated as previously reported [66,67,68].

### 4.11. In Vitro Transcription and Translation Assay

pFK I-389 plasmid containing HCV RNA (genotype 1b) was linearized by ScaI, and was used as the template for in vitro transcription. HCV mRNA was generated by MEGAscript kit containing T7 RNA polymerase (Ambion, Austin, TX, USA). In vitro transcribed mRNAs were purified by MEGAclear kit (Ambion). The integrity of all the in vitro transcribed mRNAs was confirmed by Agilent Bioanalyzer. The purified mRNAs were used for in vitro translation experiments by using the rabbit reticulocyte lysate system (Promega, Madison, WI, USA).

### 4.12. Statistical Analysis

Results are from at least three independent experiments and presented as mean ± SD. Statistical significance was estimated by student′s *t* test by GraphPad software. *p* value < 0.05 was considered statistical significant.

## Figures and Tables

**Table 1:** Herbs determined in the present study. Impact on HCV replication was evaluated in Huh-luc/neo-ET cells for 72 h and determined following the methods described in the Materials and Methods section.

Classification	Latin Name	Organ/Channel	Huh-Luc/Neo-ET	
			Anti-HCV Activity (100 μg/mL)	Cytotoxicity (100 μg/mL) or IC_50_
Drug tonify and nourish the yin	*Fructus lycii* (fruit)	Liver, Kidney, heart	N.D. ^a^	N.D.
Tonic drug	*Radix astragali* (root)	Spleen, stomach, middle burner, Lung	N.D.	N.D.
Drug tonify yang	*Rubus chingii Hu* (fruit)	Liver, Kidney	N.D.	N.D.
Drug dispel hot heat	*Flos chrysanthemi Indici* (flower)	Lung, Liver	N.D.	N.D.
Drug dispel hot heat	*Cassia obtusifolia* (seed)	Liver and Large Intestine	N.D.	JMZ-E25: 102.6 ± 2.5 μg/mL ^b^; JMZ-E75: 121.5 ± 7.9 μg/mL
Drug dispel hot heat	*Lonicera japonica* (*L. japonica*) *Thunb.* (flower)	Lung, heart, stomach, large intestine, liver	N.D.	N.D.
Drug dispel hot heat	*Forsythia suspense* (Thunb.) Vahl (fruit)	Heart, liver, gallbladder, small intestine	N.D.	N.D.
Drug promote blood circulation	*Poria cocos* (Schw.) Wolf) (sclerotia)	Heart, Kidney, Spleen, Lung, stomach	N.D.	N.D.
Drug promote blood circulation	*Carthamus tinctorius* L. (flower)	Heart, Liver, Kidney;	N.D.	N.D.
Drug promote blood circulation	*Spatholobus suberectus* (stem)	Liver and Kidney	JXT JXT-E0 JXT-E25 JXT-E50 JXT-E75	JXT-E0: 89.1 ± 9.4 μg/mL
Drug promote blood circulation	*Leonurus japonicas* Houtt. (leaf)	Liver, heart kidney and Pericardium	N.D.	N.D.
Drug regulate and tonify the blood, alleviate food stagnation	*Crataegus pinnatifida Bge.* (fruit)	Spleen, stomach and liver	N.D.	N.D.

^a^ N.D.: not detectable. ^b^ JMZ-E25: 102.6 ± 2.5 μg/mL: the 25% ethanol eluted fraction of JMZ with IC_50_ of 102.6 ± 2.5 μg/mL.

**Table 2:** EC_50_ and IC_50_ of *S. suberectus.* fractions and approved anti-HCV therapy on Huh-luc/neo-ET cells. Impact on HCV replication and cytotoxicity were evaluated in Huh-luc/neo-ET cells for 72 h following the methods described in the Materials and Methods section.

Fractions	Anti-HCV Activity (EC_50_) ^a^	Cytotoxicity (IC_50_) ^b^
JXT	1.68 ± 0.39 μg/mL	>100 μg/mL
JXT-E0	74.1 ± 1.0 μg/mL	89.1 ± 9.4 μg/mL
JXT-E25	0.45 ± 0.08 μg/mL	>100 μg/mL
JXT-E50	1.03 ± 0.06 μg/mL	>100 μg/mL
JXT-E75	1.13 ± 0.15 μg/mL	>100 μg/mL
Telaprevir	185.0 ± 35.0 nM (125.77 ± 23.76 ng/mL)	
Sofosbuvir	26.3 ± 5.5 nM (13.94 ± 2.92 ng/mL)	
Daclatasvir	0.008 ± 0.001 nM (5.911 ± 0.522 pg/mL)	
Simeprevir	1.27 ± 0.25 nM (42.50 ± 3.90 ng/mL)	
IFNα-2a	0.760 ± 0.115 fg/mL	

^a^ Detected by Luciferase report assay as described in Materials and Methods after 72 h treatment; ^b^ Detected by Methylene blue assay after 72 h treatment.

**Table 3:** Content of polyphenols and tannins in the crude water extract of *S. suberectus* and ethanol fractions from macroporous resin.

Fraction	ECG (%)	EGCG (%)	EC (%)	EGC (%)	Gallic Acid (%)	(+)-Catechin (%)	Tannin (Mean ± SD, %) (*n* = 3)
JXT	N.D. *	0.01	1.08	0.59	0.01	0.34	20.2 ± 1.1
JXT-E0	N.D. *	N.D. *	0.09	0.03	N.D. *	0.01	0.2 ± 1.9
JXT-E25	N.D. *	0.01	N.D. *	0.12	0.04	0.04	0.3 ± 1.3
JXT-E50	N.D. *	0.01	2.10	1.17	N.D. *	0.69	27.0 ± 1.6
JXT-E75	0.04	0.10	0.39	0.03	0.01	0.01	20.2 ± 1.2

* N.D., not detected.

**Table 4:** Anti-HCV activity of major components of *S. suberectus*. The anti-HCV activity and cytotoxicity were evaluated in Huh-luc/neo-ET cells for 72 h treatment and determined as described in the Materials and Methods Section. The results were presented as mean ± S.D. from at least three separate experiments.

Compound	CID	Anti-HCV Activity (EC_50_, 72 h)	Cytotoxicity (IC_50_, 72 h)
ECG	107905	69.833 ± 5.620 μM	>100 μM
EGCG	65064	68.667 ± 4.509 μM	>100 μM
EC	72276	>100 μM	>100 μM
EGC	72277	>100 μM	>100 μM
Gallic acid	370	>100 μM	>100 μM
(+)-Catechin	9064	>100 μM	>100 μM

## Abbreviations

ARDS: Acute respiratory distress syndrome
CHX: Cycloheximide
*C. pinnatifida Bge*: *Crataegus pinnatifida Bge*
*C. obtusifolia*: *Cassia obtusifolia*
*C. tinctorius* L.: *Carthamus tinctorius* L.
DAAs: Direct anti-viral agents
EC: Epicatechin
ECG: Epicatechin gallate
EGC: Epigallocatechin
EGCG: Epigallocatechin gallate
FBS: Fetal bovine serum
*F. chrysanthemi Indici*: *Flos chrysanthemi Indici*
*F. lycii*: *Fructus Lycii*
*F. suspense Thunb*: *Forsythia suspense (Thunb.) Vahl*
HCV: Hepatitis C virus
JXT: *Spatholobus suberectus*
*L. japonicus Houtt*: *Leonurus japonicas Houtt*
*L. japonica Thunb*: *Lonicera japonica Thunb*
MNV-1: Murine norovirus-1
NEAA: Non-essential amino acid
NS proteins: Nonstructural proteins
*P. cocos*: *Poria cocos (Schw.) Wolf*
pegIFNα: Pegylated interferon α
qRT-PCR: Quantitative real-time-PCR
*R. chingii Hu*: *Rubus chingii Hu*
RT: Retention time
*S. suberectus* or *R. astragali*: *Radix astragali*
SVR: Sustained viral response
